# New Dammarane-Type Saponins from *Gynostemma pentaphyllum*

**DOI:** 10.3390/molecules24071375

**Published:** 2019-04-08

**Authors:** Po-Yen Chen, Chih-Chao Chang, Hui-Chi Huang, Li-Jie Zhang, Chia-Ching Liaw, Yu-Chi Lin, Nham-Linh Nguyen, Thanh-Hoa Vo, Yung-Yi Cheng, Susan L. Morris-Natschke, Kuo-Hsiung Lee, Yao-Haur Kuo

**Affiliations:** 1 Division of Chinese Materia Medica Development, National Research Institute of Chinese Medicine, Taipei 112, Taiwan; peik35@gmail.com (P.-Y.C.); changhcym@gmail.com (C.-C.C.); lijizhang@hotmail.com (L.-J.Z.); m8952612@hotmail.com (Y.-C.L.); nguyenlinhnham4201@gmail.com (N.-L.N.); hoavo0808@gmail.com (T.-H.V.); 2 Department of Life Sciences and Institute of Genome Sciences, National Yang-Ming University, Taipei 112, Taiwan; 3 Department of Chinese Pharmaceutical Sciences and Chinese Medicine Resources, China Medical University, Taichung 404, Taiwan; hchuang@mail.cmu.edu.tw; 4 Department of Research and Development, Starsci Biotech Co. Ltd., Taipei 112, Taiwan; biogodas@hotmail.com; 5 The Ph.D. Program in Clinical Drug Development of Chinese Herbal Medicine, College of Pharmacy, Taipei Medical University, Taipei 11031, Taiwan; 6 Natural Products Research Laboratories, Eshelman School of Pharmacy, University of North Carolina, Chapel Hill, NC 27599-7568, USA; yungyi@email.unc.edu (Y.-Y.C.); susan_natschke@unc.edu (S.L.M.-N.); 7 Chinese Medicine Research and Development Center, China Medical University and Hospital, Taichung 40402, Taiwan; 8 Graduate Institute of Integrated Medicine, College of Chinese Medicine, China Medical University, Taichung 404, Taiwan

**Keywords:** *Gynostemma pentaphyllum*, Jiaogulan, dammarane-type saponins, gypenosides antiproliferative activity

## Abstract

Six new dammarane-type saponins, gypenosides CP1-6 (**1**–**6**), along with 19 known compounds **7**–**25**, were isolated and characterized from the aerial parts of *Gynostemma pentaphyllum*. Among these compounds, eight dammarane-type saponins, **2**, **5**, **6**, **7**, **11**, **12**, **13**, and **15,** exhibited the greatest antiproliferative effects against two human tumor cell lines (A549 and HepG2).

## 1. Introduction

*Gynostemma pentaphyllum* (Thunb.) Makino (family Cucurbitaceae) is an ethnomedicine frequently used in Asian countries as a functional food and tea [1,2,3,4]. Due to its various pharmacological activities, including anti-inflammatory [5,6,7], antioxidative [8,9], anti-hyperlipidemic [3,10], hypoglycemic [3,11,12], and antitumor effects [13,14,15,16,17,18], it is marketed in Asia in dietary supplements, such as Jiaogulan tea and Jiaogulan concentrated juice [4,12,19]. Dammarane-type triterpene saponins or gypenosides are the major components responsible for the plant’s pharmacological activities [1,2,3,4,5,6,7,11,15,17,20,21,22,23,24,25]. To date, more than 210 compounds, including over 180 gypenosides, along with flavonoids [9,15,17] and polysaccharides [8,16] have been isolated from *G. pentaphyllum.* Moreover, gypenosides are structurally like the ginsenosides, which are well-known pharmacologically active components of ginseng root (*Panax ginseng*) [26]. Thus, *G. pentaphyllum* is a unique non-Panax plant rich in gypenosides [26,27].

**Figure 1:**
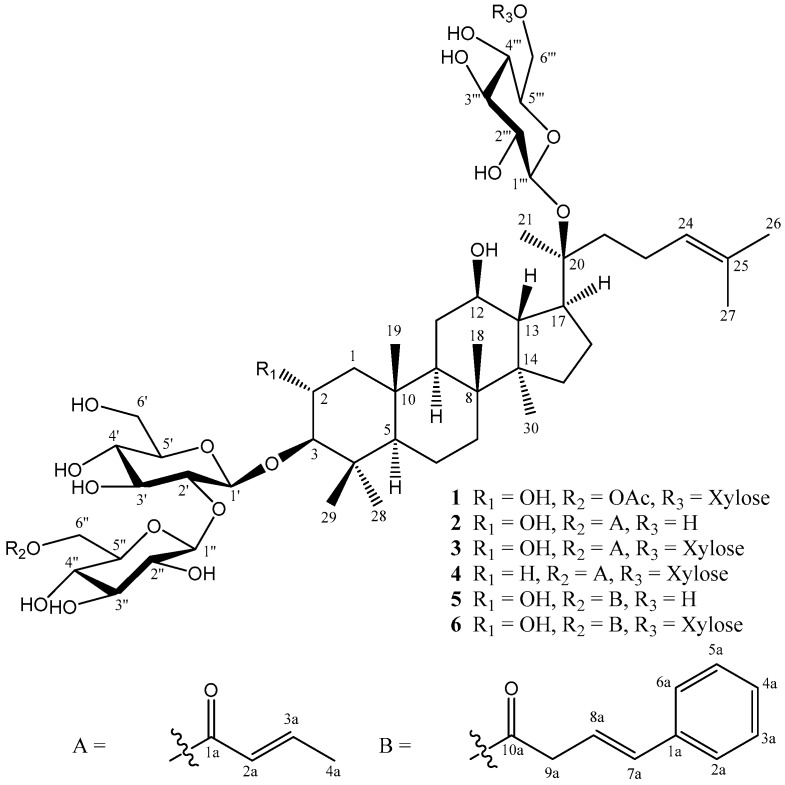
Chemical structures of compounds **1**–**6** isolated from *G. pentaphyllum*.

In our present study, 25 components were isolated from an ethanol extract of the aerial parts of *G. pentaphyllum.* Their structures were identified from NMR, IR and HRMS spectroscopic data. Among them, six new gypenosides CP1-6 (**1**–**6**) (Figure 1) and 10 known dammarane-type saponins **7**–**16**, seven known flavonoid glycosides **17**–**23**, and two known sesquiterpene glycosides **24** and **25**, were obtained from the title plant. All isolated compounds were evaluated for antiproliferative activities against human lung cancer (A549) and hepatoma (HepG2) cell lines.

## 2. Results and Discussion

**Figure 2:**
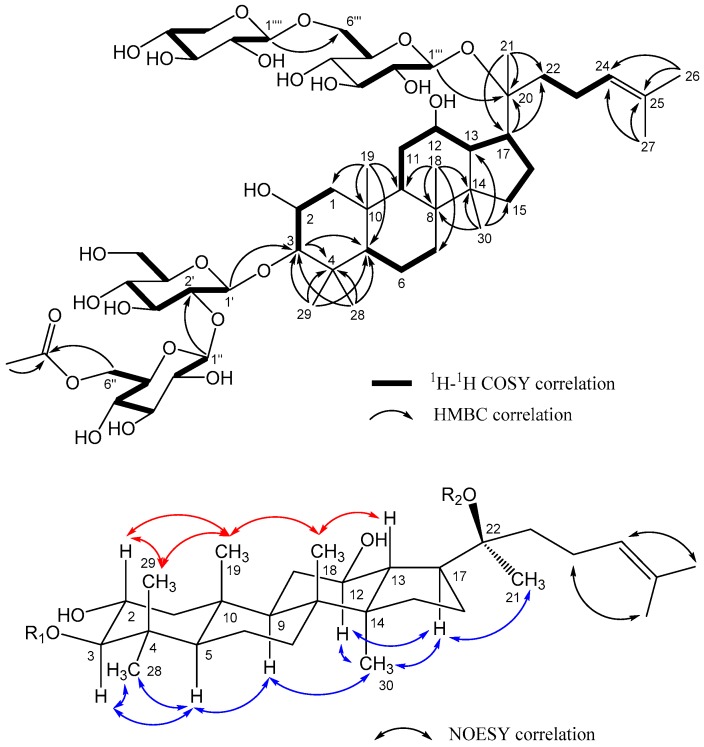
Key COSY, HMBC, and NOESY correlations of **1**.

Gypenoside CP1 (**1**), [α]^26^_D_ +11.5 (*c* 0.2, MeOH), has the molecular formula C_55_H_92_O_24_, as established by NMR and HRESIMS (*m*/*z* 1159.5874 [M + Na]^+^, calcd for C_55_H_92_O_24_Na 1159.5876), indicating ten degrees of unsaturation. The IR spectrum showed absorption bands for hydroxyl, carbonyl, and olefinic groups at 3358, 1736, and 1638 cm^−1^, respectively. In the ^1^H-NMR spectrum (Table 1 and Table 2), signals were observed for nine tertiary methyl groups [δ_H_ 0.86, 0.92, 0.97, 1.00, 1.10, 1.36, 1.62, 1.68, and 2.04 (each 3H, s)] and an olefinic proton [δ_H_ 5.13 (1H, bt, *J* = 7.0 Hz)]. The ^13^C-NMR (Table 1 and Table 2) and DEPT spectra showed resonances for 55 carbons, among which 30 were aglycone carbons including 8 methyls [δ_C_ 16.3 (C-18), 17.4 (C-30), 17.7 (C-29), 17.8 (C-19), 18.0 (C-27), 22.4 (C-21), 25.9 (C-26), 28.6 (C-28)], 4 oxygenated carbons [δ_C_ 68.2 (C-2), 71.5 (C-12), 84.9 (C-20), 96.5 (C-3)], and a pair of olefinic carbons [δ_C_ 126.1 (C-24), 132.2 (C-25)]. The ^1^H-^1^H COSY and HMBC correlations fully established the planar structure of **1** (Figure 2). Furthermore, oxygenations at C-2, C-3, and C-12 were corroborated by ^1^H-^1^H COSY correlations between H_2_-1 (δ_H_ 2.09; 0.87)/H-2 (δ_H_ 3.72)/H-3 (δ_H_ 2.95) and H-9 (δ_H_ 1.48)/H_2_-11 (δ_H_ 1.28; 1.82)/H-12 (δ_H_ 3.74)/H-13 (δ_H_ 1.73)/H-17 (δ_H_ 2.28) /H_2_-16 (δ_H_ 1.33; 1.89)/H_2_-15 (δ_H_ 1.03; 1.57) as well as the HMBC long-range correlation of H-3 with carbon signals at δ_C_ 41.8 (C-4) and 57.2 (C-5). The molecular formula and 1D and 2D-NMR spectroscopic data of **1** suggested a dammarane-type saponin, a typical constituent of *Gynostemma* species, with the same aglycone as that of 2α,3β,12β,20(*S*)-tetrahydroxydammar-24-ene [28,29]. The aglycone accounted for 30 carbon signals, leaving 25 carbon signals assignable to four sugar moieties and one acetyl group [δ_C_ 20.9 (CH_3_) and 172.8 (C=O)] in the ^13^C NMR spectrum. Four anomeric signals were observed at δ_H_ 4.30 (d, *J* = 7.5 Hz)/δ_C_ 105.5, δ_H_ 4.42 (d, *J* = 7.5 Hz)/δ_C_ 104.7, δ_H_ 4.56 (d, *J* = 8.0 Hz)/δ_C_ 98.1, and δ_H_ 4.71 (d, *J* = 7.5 Hz)/δ_C_ 105.0. HMBC cross peaks of H-1′ to C-3, H-1″ to C-2′, H-1‴ to C-20, and H-1″″ to C-6‴ determined the positions of the four sugars. Acid hydrolysis of **1** yielded d-glucose (Glc) and d-xylose (Xyl) in a ratio of 3:1 based on HPLC analysis of the component monosaccharides compared with the standard sugars [30]. A long-range correlation between proton and carbon signals at δ_H_ 4.15, 4.31 (H-6″) and δ_C_ 172.8 (C=O), respectively, was consistent with acetylation of the 6″-OH. In the NOESY spectrum of **1** (Figure 2), cross peaks were found between H-2 (δ_H_ 3.72)/Me-29 (δ_H_ 1.10), Me-29 (δ_H_ 1.10)/Me-19 (δ_H_ 0.97), Me-19 (δ_H_ 0.97)/Me-18 (δ_H_ 1.00), and Me-18 (δ_H_ 1.00)/H-13 (δ_H_ 1.73), indicating β-orientations of Me-29, Me-19, Me-18, and H-13. However, the NOESY correlations of H-3 (δ_H_ 2.95)/Me-28 (δ_H_ 0.86), H-3 (δ_H_ 2.95)/H-5 (δ_H_ 0.84), H-5 (δ_H_ 0.84)/H-9 (δ_H_ 1.48), H-9 (δ_H_ 1.48)/Me-30 (δ_H_ 0.92), and Me-30 (δ_H_ 0.92)/H-17 (δ_H_ 2.28) suggested α-orientations of H-5, H-9, H-17, Me-28, and Me-30. The configuration of C-20 in **1** was determined to be *S* based on a comparison of the ^13^C-NMR spectroscopic data of **1** and gypenoside XLVI [29]. The complete structure of **1** (gypenoside CP1) was elucidated as 2α,3β,12β,20*S*-tetrahydroxydammar-24-ene-3-*O*-[(6-*O*-acetyl-β-d-glucopyranosyl)-(1→2)-β-d-glucopyranosyl]-20-*O*-[β-d-xylopyranosyl-(1→6)-β-d-glucopyranoside].

**Figure 3:**
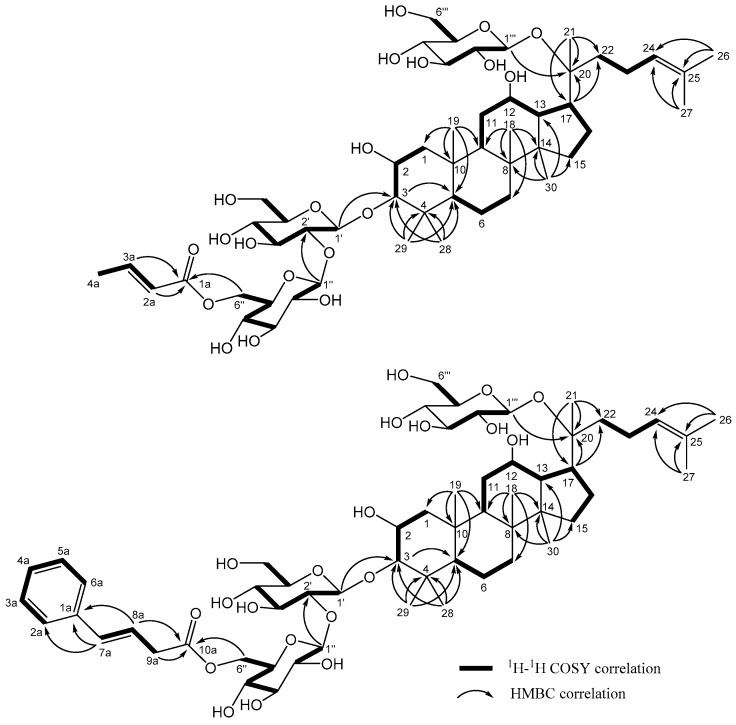
Key COSY and HMBC correlations of **2** and **5**.

Gypenoside CP2 (**2**) was isolated as a white powder. Its molecular formula was determined to be C_52_H_86_O_20_ from HRESIMS and ^13^C-NMR spectroscopic analysis. Comparison of the ^1^H and ^13^C-NMR spectroscopic data of **1** and **2** (Table 1 and Table 2) indicated that both compounds have the same aglycone; however, compound **2** contains a but-2-enoyl unit (δ_H_ 7.00, 5.88, and 1.89/δ_C_ 168.1, 146.6, 123.6, and 18.1) but lacks the xylose and acetyl group found in **1**. The location of the but-2-enoyl group at Glc-C-6″ was confirmed by the correlations observed in the HMBC between δ_H_ 4.18, 4.37 (H-6″) and δ_C_ 168.1 (C-1a, C=O) (Figure 3). The NMR spectra showed the presence of three β-glucopyranosyl signals [δ_H_ 4.42 (d, *J* = 7.5 Hz)/δ_C_ 104.6, δ_H_ 4.71 (d, *J* = 7.5 Hz)/δ_C_ 105.2, δ_H_ 4.60 (d, *J* = 8.0 Hz)/δ_C_ 98.3], which were confirmed to be from d-glucose via acid hydrolysis. Long-range correlations were also observed between δ_H_ 4.42 (H-1′) and δ_C_ 96.5 (C-3), δ_H_ 4.71 (H-1″) and δ_C_ 82.7 (C-2′), and δ_H_ 4.60 (H-1‴) and δ_C_ 84.9 (C-20) indicating that the three sugars were attached to C-3, C-2′, and C-20, respectively. Thus, the structure of gypenoside CP2 (**2**) was elucidated as 2α,3β,12β,20*S*-tetra-hydroxydammar-24-ene-3-*O*-{[6-*O*-(*E*)-but-2-enoyl]-β-d-glucopyranosyl-(1→2)-β-d-glucoyranosyl}-20-*O*-β-d-glucopyranoside.

Gypenoside CP3 (**3**) was isolated as a white powder. Its molecular formula was determined as C_57_H_94_O_24_ from HRESIMS and ^13^C-NMR spectroscopic analysis. The ^1^H- and ^13^C-NMR spectra (Table 1 and Table 2) of **3** showed signals assignable to a 3-*O*-{[6-*O*-(*E*)-but-2-enoyl]-β-d-glucopyranosyl-(1→2)-β-d-glucopyranosyl} moiety and a 20-*O*-[β-d-xylopyranosyl-(1→6)-β-d-glucopyranosyl] moiety, which were virtually superimposable onto those of **2**; however, carbon signals (δ_C_ 66.8, 71.2, 74.8, 77.5, and 105.6) consistent with an additional sugar moiety were also present. Four anomeric signals were found at δ_H_ 4.29 (d, *J* = 7.2 Hz)/δ_C_ 105.6, δ_H_ 4.41 (d, *J* = 7.8 Hz)/δ_C_ 104.6, δ_H_ 4.56 (d, *J* = 7.8 Hz)/δ_C_ 98.1, and δ_H_ 4.70 (d, *J* = 7.8 Hz)/δ_C_ 105.2. Acid hydrolysis of **3** yielded d-glucose and d-xylose (3:1). The signals for CH_2_-6‴ (δ_H_ 3.72, 4.00/δ_C_ 70.1) in **3** were shifted downfield compared to those in **2** (δ_H_ 3.64, 3.77/δ_C_ 62.5), indicative of the attachment of a d-xylose at CH_2_-6‴ in **3 [29]**. Moreover, long-range correlations (HMBC) between δ_H_ 4.41 (H-1′) and δ_C_ 96.5 (C-3), δ_H_ 4.70 (H-1″) and δ_C_ 82.7 (C-2′), δ_H_ 4.56 (H-1‴) and δ_C_ 84.9 (C-20), and δ_H_ 4.29 (H-1″″) and δ_C_ 70.1 (C-6‴) indicated the following sugar locations, d-glucose at C-3, C-2′, and C-20, respectively and d-xylose at C-6‴. Accordingly, compound **3** (gypenoside CP3) was determined as 2α,3β,12β,20*S*-tetrahydroxydammar-24-ene-3-*O*-{[6-*O*-(*E*)-but-2-enoyl]-β-d-glucopyranosyl-(1→2)-β-d-glucopyranosyl}-20-*O*-[β-d-xylopyranosyl-(1→6)-β-d-glucopyranoside].

Gypenoside CP4 (**4**) was isolated as a white powder. The HRESIMS and ^13^C-NMR spectroscopic data of **4** suggested its molecular formula to be C_57_H_94_O_23_. Analysis of the ^1^H- and ^13^C-NMR spectra (Table 1 and Table 2) gave 57 signals, of which 30 were assigned to the triterpene skeleton. The further comparison of the 1D and 2D NMR data of **3** and **4** indicated the structural similarity in a 3β,12β,20*S*-trihydroxydammar-24-ene with four sugar moieties, except for the replacement of an oxymethine (δ_C_ 68.2, C-2) by a methylene (δ_C_ 27.3, C-2) at aglycone in **4**. Detailed checking the NMR data together with the analysis of acid hydrolysis, the glycone moiety of **4** were composed 3 units of of d-glucose and one d-xylose. Thus, gypenoside CP4 was determined as 3β,12β,20*S* -trihydroxydammar-24-ene-3-*O*-{[6-*O*-(*E*)-but-2-enoyl]-β-d-glucopyranosyl-(1→2)-β-d-glucopyranosyl}-20-*O*-[β-d-xylopyranosyl-(1→6)-β-d-glucopyranoside].

The HRESIMS of gypenoside CP5 (**5**) showed a quasimolecular ion at *m/z* 1129.5928 [M + Na]^+^ (calcd. for C_58_H_90_O_20_Na 1129.5923), corresponding to the molecular formula C_58_H_90_O_20_. Like previous isolates, compound **5** has a 2α,3β,12β,20 (*S*)-tetrahydroxydammar-24-ene skeleton, due to the similarity of the ^1^H- and ^13^C-NMR spectroscopic data (Table 1 and Table 2). Detailed analysis of the NMR and HRESIMS data of **5** and **2**, suggested that both compounds possess the same aglycone and d-glucopyranosyl moieties, while compound **5** contains a phenyl moiety not found in **2**. The cross peaks between δ_H_ 4.20, 4.38 (H-6″) and δ_H_ 173.4 (C-10a) in the HMBC spectrum of **5** (Figure 3) indicated that the (E)-but-2-enoyl ester at Glc C-6″ in **2** was replaced by a (*E*)-4-phenylbut-3-enoyl unit in **5**. Accordingly, the structure of **5** (gypenoside CP5) was confirmed as 2α,3β,12β,20*S*-tetrahydroxydammar-24-ene-3-*O*-{[6-*O*-(*E*)-4-phenyl-but-3-enoyl]-β-d-glucopyranosyl-(1→2)-β-d-glucopyranosyl}-20-*O*-β-d-glucopyranoside.

The positive HRESIMS of compound **6** showed a molecular ion peak at *m/z* 1261.6340 [M + Na]^+^ (calcd for C_63_H_98_O_24_Na, 1261.6346), which was 132 amu more than the molecular ion of **5**, presumably corresponding to a xylose group. The 1D and 2D NMR spectroscopic data of **6** showed similar signals as those of **5** except for an additional unit in **6** characterized by a xylose signals (δ_C_ 105.6, 77.5, 74.8, 71.2, and 66.8). Acidic hydrolysis of **6** also furnished d-glucose and d-xylose. Based on the above corroborations, the structure of **6** (gypenoside CP6) was determined as 2α,3β,12β,20*S*-tetrahydroxydammar-24-ene-3-*O*-{[6-*O*-(*E*)-4-phenyl-but-3-enoyl]-β-d-glucopyranosyl-(1→2)-β-d-glucopyranosyl}-20-*O*-[β-d-xylopyranosyl-(1→6)-β-d-glucopyranoside].

After the detailed spectroscopic analysis and chemical hydrolysis mentioned above, compounds **1**-**6** were proved as novel chemical structures and named as gypenosides CP1-CP6, respectively. The remaining nineteen isolates were identified as 2α,3β,12β,20*S*-tetrahydroxydammar-24-ene-3-*O*-β-d-glucopyranosyl-20-*O*-[β-d-6-*O*-acetylglucopyranosyl-(1→2)-β-d-glucopyranoside (**7**) [31], gypenoside XLVI (**8**) [29], gypenoside LVI (**9**) [29], gypenoside LVII (**10**) [32], gypenoside LXXVII (**11**) [33], gypenoside L (**12**) [29], 2α,3β,12β,20*S*-tetrahydroxydammar-24-ene-3-*O*-β-d-glucopyranosyl-20-*O*-β-d-glucopyranoside (**13**) [34], gypenoside XLII (**14**) [35], gypenoside Rd (**15**) [36] and 2α,3β,20*S*-trihydroxydammar-24-ene-3-*O*-[β-d-glucopyranosyl-(1→2)-β-d-glucopyranosyl]-20-*O*-[β-d-xylo-pyranosyl-(1→6)-β-d-glucopyranoside] (**16**) [37], together with seven flavonoids, quercetin-3-*O*-α-l-rhamnopyranosyl(1→2)-β-d-galactopyranoside (**17**) [38], quercetin-3-neohesperidoside (**18**) [39], kaempferol-3-*O*-α-l-rhamnopyranosyl-(1→2)-β-d-galactopyranoside (**19**) [40], kaempferol-3-*O*-α-l-rhamnopyranosyl-(1→2)-β-d-glucopyranoside (**20**) [41], quercetin-*7*-*O*-β-d-glucoside (**21**) [42], kaempferol-7-*O*-β-d-galactopyranoside (**22**) [43], and isorhamnetin-7-*O*-β-d-glucopyranoside (**23**) [44], and two sesquiterpene glucosides, (6*R*,7*E*,9*R*)-9-hydroxy-megastigman-4,7-dien-3-one-9-*O*-β-d-glucopyranoside (**24**) [45], and (*E*)-4-[3′-(β-d-glucopyranosyloxy)butylidene]-3,5,5-trimethyl-2-cyclohexen-1-one (**25**) [46]. The structures of the known compounds were identified by comparing their NMR data with published literature.

All isolates **1**–**25** were evaluated for antiproliferative activities against two human tumor cell lines, adenocarcinoma (A549) and human liver carcinoma (HepG2) and the results are shown in Table 3. Although none of the isolates showed significant cytotoxcity against the two human cell lines, certain dammarane-type triterpene saponins (**2**, **5**, **6**, **7**, **11**, **12**, **13**, and **15**) were more potent than the remaining compounds. The EC_50_ values of these eight compounds against the HepG2 cell line ranged from 29.3 to 100.6 μM, while only four compounds (**2**, **11**, **13** and **15**) exhibited EC_50_ values of less than 100 μM (EC_50_ 59.4~87.3 μM) against A549 cells. Among the six new gypenosides, compound **2** was the most potent against A549 cells and compound **5** was among the most potent against HepG2 cells.

## 3. Materials and Methods

### 3.1. General Experimental Procedures

The optical rotations were determined using a JASCO P-2000 polarimeter (Jasco Co., Tokyo, Japan). The infrared (IR) spectra were measured on a Mattson Genesis II spectrophotometer (Thermo Fisher Scientific Inc., Waltham, MA, USA). Electrospray ionization mass spectrometry (ESIMS) data were obtained on an LCQ mass spectrometer (Thermo Fisher Scientific Inc., Waltham, MA, USA). High-resolution electronic ionization mass spectrometry (HREIMS) data were measured on a Finnigan MAT-95XL mass spectrometer (Thermo Fisher Scientific Inc., Waltham, MA, USA). Nuclear magnetic resonance (NMR) spectra were recorded using by Bruker AC-400 FT-NMR (Bruker BioSpin, Rheinstetten, Germany), Varian unit Inova 500 MHz, and Varian VNMRS 600 MHz spectrometers (Aglient Technologies, Santa Clara, CA, USA). Diaion HP-20 (Mitsubishi Chemical Co., Tokyo, Japan), Sephadex LH-20 (GH Healthcare, Uppsala, Sweden), and silica gel 60 (Merck 70–230 and 230–400 mesh, Merck, Darmstadt, Germany) were used for column chromatography, and precoated silica gel (Merck 60 F-254) plates were used for TLC. The spots on TLC were detected by spraying with an anisaldehyde-sulfuric acid solution and heating at 100 °C. HPLC separations were performed on a Shimadzu LC-6AD series instrument (Shimadzu Inc., Kyoto, Japan) with an SPD-10A UV detector and a 380-LC ELSD detector (Aglient Technologies, Santa Clara, CA, USA), which was equipped with a Cosmosil 5C_18_ AR-II column (Nacalai Tesque, Inc., Kyoto, Japan).

### 3.2. Plant Material

The aerial parts of *Gynostemma pentaphyllum* (8.4 kg) were purchased from Zheng Yuen Tang Biotech Co. Ltd. in Kaohsiung, Taiwan in August 2013. A voucher specimen (NRICM, No. 20130101) has been deposited in the National Research Institute of Chinese Medicine, Taipei, Taiwan.

### 3.3. Extraction and Isolation

The dried aerial parts of *G. pentaphyllum* (8.4 kg) were extracted three times at 60 °C with 95% ethanol (EtOH). The EtOH soluble portion was concentrated to give a crude extract (8 L). The concentrated EtOH extract was partitioned with *n*-hexane and H_2_O (1:1, *v*/*v*) to give a *n*-hexane portion (847.2 g). The aqueous layer was further partitioned with EtOAc to give an EtOAc portion (279.5 g). Then, the H_2_O portion was loaded onto a Diaion HP-20 column (11 × 72 cm), and successively eluted with H_2_O, 25% MeOH, 50% MeOH, 75% MeOH, 100% MeOH, and 100% EtOAc to obtain five fractions (Fr-1 to 6). Fr-4 (298.7 g) was further chromatographed on a Sephadex LH-20 column with 60% MeOH as the eluent to give four fractions (Fr-4-1 to Fr-4-4). Fr-4-3 was further purified by semi-preparative HPLC using 35% CH_3_CN in H_2_O as the solvent system at a flow rate of 2.0 mL/min to give compounds **1** (71.5 mg), **7** (30.3 mg), **8** (54.2 mg), **9** (32.1 mg), and **16** (30.4 mg). Fr-3 was chromatographed on a LH-20 column with 60% MeOH as the eluent to yield eight fractions (Fr-3-1 to Fr-3-8). Fr-3-2 and Fr-3-3 were further separated by semi-preparative HPLC with 35%, and 18% CH_3_CN in H_2_O as the solvent system, respectively. Compounds **24** (17.2 mg) and **25** (5.4 mg) were obtained from Fr-3-3, and compound **14** (15.4 mg) was separated from Fr-3-2. Fr. 5 was further fractioned with a step gradient elution of H_2_O-MeOH (from 30:70 to 0:100, *v*/*v*) on a C_18_-gel flash column to afford six fractions (Fr-5-1 to Fr-5-6). Fr-5-2, and Fr-5-3 were further purified by semi-preparative HPLC using 38% CH_3_CN in H_2_O as the solvent system at a flow rate of 2.0 mL/min to give compounds **2** (5.2 mg), **3** (3.1 mg), **4** (2.8 mg), and **10** (4.5 mg). Fr-5-4, was further purified by semi-preparative HPLC using 48% CH_3_CN in H_2_O as the solvent system at a flow rate of 2.0 mL/min to give compounds **5** (2.6 mg), **6** (2.1 mg), **12** (11.2 mg), and **13** (7.9 mg). The EtOAc portion was loaded onto a LH-20 column eluting with CH_2_Cl_2_/MeOH (1:1, *v*/*v*) to afford 10 fractions (Fr-E-1 to Fr-E-10). Fr-E-2 was further purified by semi-preparative HPLC using 80% CH_3_CN in H_2_O as the solvent system at a flow rate of 2.0 mL/min to give compound **11** (8.7 mg). Fr-E-8 was further purified by semi-preparative HPLC using 38% CH_3_CN in H_2_O as the solvent system at a flow rate of 2.0 mL/min to give compounds **17** (13.7 mg), **18** (42.7 mg), **19** (30.2 mg), **20** (48.0 mg), **21** (7.0 mg), **22** (4.2 mg), and **23** (16.2 mg).

### 3.4. Spectroscopic Data (^1^H- and ^13^C-NMR spectra of **1**-**6** were also provided by the supplementary materials)

*Gypenoside CP1* (**1**), White amorphous powder; [α]^26^_D_ +11.5 (*c* 0.2, MeOH); IR (KBr) ν_max_ 3358, 2945, 2876, 1736, 1638, 1082 cm^−1^; ^1^H- (500 MHz, methanol-*d*_4_) and ^13^C- (125 MHz, methanol-*d*_4_) NMR data, see Table 1 and Table 2, respectively; HRESIMS *m*/*z* 1159.5874 [M + Na]^+^ (calcd for C_55_H_92_O_24_Na, 1159.5876).

*Gypenoside CP2* (**2**), White amorphous powder; [α]^26^_D_ +15.7 (*c* 0.2, MeOH); IR (KBr) ν_max_ 3362, 2941, 2872, 1715, 1650, 1074 cm^−1^; ^1^H- (500 MHz, methanol-*d*_4_) and ^13^C- (125 MHz, methanol-*d*_4_) NMR data, see Table 1 and Table 2, respectively; HRESIMS *m*/*z* 1053.5607 [M + Na]^+^ (calcd for C_52_H_86_O_20_Na, 1053.5610).

*Gypenoside CP3* (**3**), White amorphous powder; [α]^26^_D_ +12.9 (*c* 0.2, MeOH); IR (KBr) ν_max_ 3370, 2928, 2876, 1715, 1650, 1078 cm^−1^; ^1^H- (600 MHz, methanol-*d*_4_) and ^13^C- (150 MHz, methanol-*d*_4_) NMR data, see Table 1 and Table 2, respectively; HRESIMS *m*/*z* 1185.6030 [M + Na]^+^ (calcd for C_57_H_94_O_24_Na, 1185.6033). 

*Gypenoside CP4* (**4**), White amorphous powder; [α]^26^_D_ +6.6 (*c* 0.2, MeOH); IR (KBr) ν_max_ 3350, 2937, 2868, 1728, 1650, 1078 cm^−1^; ^1^H- (600 MHz, methanol-*d*_4_) and ^13^C- (150 MHz, methanol-*d*_4_) NMR data, see Table 1 and Table 2, respectively; HRESIMS *m*/*z* 1169.6084 [M + Na]^+^ (calcd for C_57_H_94_O_23_Na, 1169.6084).

*Gypenoside CP5* (**5**), White amorphous powder; [α]^26^_D_ +7.7 (*c* 0.2, MeOH); IR (KBr) ν_max_ 3370, 2921, 2872, 1679, 1074 cm^−1^; ^1^H- (600 MHz, methanol-*d*_4_) and ^13^C- (150 MHz, methanol-*d*_4_) NMR data, see Table 1 and Table 2, respectively; HRESIMS *m*/*z* 11129.5928 [M + Na]^+^ (calcd for C_58_H_90_O_20_Na, 1129.5923).

*Gypenoside CP6* (**6**), White amorphous powder; [α]^26^_D_ +12.9 (*c* 0.2, MeOH); IR (KBr) ν_max_ 3370, 2921, 2864, 1687, 1070 cm^−1^; ^1^H- (600 MHz, methanol-*d*_4_) and ^13^C- (150 MHz, methanol-*d*_4_) NMR data, see Table 1 and Table 2, respectively; HRESIMS *m*/*z* 1261.6340 [M + Na]^+^ (calcd for C_63_H_98_O_24_Na, 1261.6346).

### 3.5. Acid Hydrolysis of Dammarane-Type Glycosides

Each isolated compound (1.0 mg) was treated with 2 N methanolic HCl (2 mL) under conditions of reflux at 90 °C for 1 h. Each mixture was extracted with CH_2_Cl_2_ to afford the aglycone portion, and the aqueous layer was neutralized with Na_2_CO_3_ and filtered. To the evaporated filtrate was added 1-(trimethylsilyl)imidazole and pyridine (0.2 mL), and the mixture was stirred at 60 °C for 5 min. After the reaction mixture was dried under a stream of N_2_, each residue was partitioned between CHCl_3_ and H_2_O. Each CH_2_Cl_2_ fraction was subjected to gas chromatography (GC, column: Varian capillary column CP-chirasil-L-val for optical isomers, 25 m × 0.25 mm, 0.12 μm; column temperature, 50–150 °C, 30 °C/min, 150–180 °C, 0.8 °C /min; injector temperature, 200 °C; He carrier gas, 2.0 kg/cm^3^; mass detector, Thermo, DSQ2; electron energy, 70 eV). Under these conditions, the sugars of each reactant were identified by comparison with authentic standards (d-glucose and d-xlyose).

### 3.6. Antiproliferation Assay

The isolates were tested for antiproliferative effects against HepG2 (human hepatocellular carcinoma), A549 (human lung adenocarcinoma) tumor cell lines and the M10 (human mammary epithelial) cell line in vitro using the MTT [3-(4,5-dimethylthiazol-2-yl)-2,5-diphenyltetrazolium bromide] colorimetric method based on previously published procedures [47]. Two cell lines were maintained optimal medium (Life Technologies) supplemented with 2 mM l-glutamine and 10% heat-inactivated fetal bovine serum (FBS) (Life Technologies) under standard culture conditions. After treatment with serial dilutions of tested compounds for 48 h, the alamar blue assay (Biosource International, Nivelles, Belgium) was used to obtain the half maximal inhibitory concentration (IC_50_). Doxorubicin was used as a positive control. Plates were incubated at 37 °C for 6 h prior to measure the absorbance at 570 nm and at 600 nm wavelengths using a spectrophotometric plate reader (DYNEX Technologies, Chantilly, VA, USA). Experimental data were normalized to control values. Mitomycin *c* was used as a positive control (A549 cell line: 0.1 ± 0.01 μg/mL; HepG2 cell line: 0.1 ± 0.01 μg/mL).

## 4. Conclusions

In this study, we isolated and characterized six new and ten known dammarane-type triterpene saponins as well as eight known flavonoids and two known sesquiterpene glucosides from a 95% EtOH extract of dried aerial parts of *G. pentaphyllum*. All the new chemical structures of compounds **1**–**6** were elucidated completely and tentatively named gypenosides CP1–CP6. These new isolates were obtained from the titled plant for the first time, and not yet found in other natural resources or synthesized molecules before. For the derived chemical structures of **5** and **6**, (*E*)-4-phenylbut-3-enyl unit is first time to be found in nature. This study adds to the present phytochemical and properties information on this plant species, together with the studies performed and compiled by others [1], could assist in future modification of dammarane-type compounds as anticancer or other therapeutic agents.

## Figures and Tables

**Table 1:** ^1^H- and ^13^C-NMR spectroscopic data of the aglycone of gypenosides CP1-6 (**1**–**6**).

No.	1		2		3		4		5		6	
	δ_H_ (mult, *J* in Hz)	δ_C_	δ_H_ (mult, *J* in Hz)	δ_C_	δ_H_ (mult, *J* in Hz)	δ_C_	δ_H_ (mult, *J* in Hz)	δ_C_	δ_H_ (mult, *J* in Hz)	δ_C_	δ_H_ (mult, *J* in Hz)	δ_C_
1	2.09 (dd, 5.0, 13.0)	47.8	2.07 (m)	47.8	2.07 (dd, 4.8, 12.6)	47.8	1.70 (m)	40.2	2.03 (dd, 4.2, 12.6)	47.7	2.03 (dd, 4.2, 12.6)	47.7
	0.87 (m)		0.91 (t, 8.0)		0.88 (m)		0.98 (m)		0.87 (m)		0.87 (m)	
2	3.72 (m)	68.2	3.70 (m)	68.2	3.69 (m)	68.2	1.68 (m); 1.95 (m)	27.3	3.70 (m)	68.2	3.70 (m)	68.2
3	2.95 (d, 9.0)	96.5	2.93 (d, 9.5)	96.5	2.93(d, 9.6)	96.5	3.11 (dd, 3.0, 9.0)	91.0	2.92 (d, 9.6)	96.6	2.92 (d, 9.6)	96.6
4	-	41.8	-	41.8		41.8		40.5	-	41.8	-	41.8
5	0.84 (d, 11.5)	57.2	0.84 (d, 10.5)	57.2	0.82 (m)	57.2	0.74 (d, 11.4)	57.6	0.79 (d, 11.4)	57.2	0.79 (d, 11.4)	57.2
6	1.49 (m); 1.56 (m)	19.3	1.46 (m); 1.54 (m)	19.4	1.46 (m); 1.53 (m)	19.4	1.43 (m); 1.53 (m)	19.3	1.37 (m); 1.50 (m)	19.4	1.37 (m); 1.50 (m)	19.4
7	1.57 (m); 1.29, (m)	35.7	1.55 (m); 1.30 (m)	35.7	1.57 (m); 1.28 (m)	35.7	1.55 (m); 1.26 (m)	35.9	1.47 (m); 1.19 (m)	35.6	1.47 (m); 1.19 (m)	35.6
8	-	41.0	-	41.0	-	41.0	-	41.0		40.9	-	40.9
9	1.48 (m)	51.0	1.50 (m)	51.0	1.47 (m)	51.0	1.42 (dd, 3.0, 13.2)	51.1	1.45 (m)	50.9	1.45 (m)	51.0
10	-	38.8	-	38.8	-	38.8		37.9	-	38.8	-	38.7
11	1.82 (m);1.28 (m)	31.0	1.86 (m); 1.28 (m)	31.1	1.82 (m); 1.28 (m)	31.0	1.78 (m); 1.22 (m)	30.8	1.81 (m); 1.22 (m)	31.1	1.81 (m); 1.22, (m)	30.9
12	3.74 (m)	71.5	3.67 (m)	71.8	3.73 (m)	71.5	3.71 (m)	71.7	3.66 (m)	71.8	3.66 (m)	71.5
13	1.73 (d, 11.5)	49.7	1.74 (d, 10.5)	49.8	1.73 (d, 10.8)	49.8	1.72 (t, 10.8)	49.7	1.70 (t, 10.8)	49.7	1.70 (t, 10.8)	49.7
14	-	52.4		52.5	-	52.4	-	52.4	-	52.5	-	52.4
15	1.57 (m); 1.03 (m)	31.5	1.58 (m); 1.06 (m)	31.6	1.58 (m); 1.04 (m)	31.5	1.57 (m); 1.03 (m)	31.5	1.55 (m); 1.03 (m)	31.6	1.55 (m); 1.03 (m)	31.5
16	1.89 (m); 1.33 (m)	27.3	1.92 (m); 1.38 (m)	27.2	1.89 (m); 1.33 (m)	27.3	1.95 (m), 1.32 (m)	27.3	1.91 (m); 1.38 (m)	27.2	1.91 (m); 1.38 (m)	27.2
17	2.28 (m)	52.9	2.27 (m)	53.1	2.28 (m)	52.9	2.28 (m)	52.9	2.28 (m)	53.1	2.28 (m)	52.9
18	1.00 (s)	16.3	1.00 (s)	16.2	0.99 (s)	16.3	0.99 (s)	16.3	0.89 (s)	16.2	0.89 (s)	16.2
19	0.97 (s)	17.8	0.94 (s)	17.8	0.93 (s)	17.8	0.88 (s)	16.8	0.84 (s)	17.9	0.84 (s)	17.9
20	-	84.9	-	84.9	-	84.9	-	85.0	-	84.9	-	84.9
21	1.36 (s)	22.4	1.34 (s)	22.8	1.35 (s)	22.4	1.35 (s)	22.4	1.33 (s)	22.9	1.33 (s)	22.4
22	1.79 (m); 1.53(m)	36.8	1.80 (m); 1.60 (m)	36.7	1.79 (m); 1.52 (m)	36.7	1.80 (m); 1.52 (m)	36.7	1.80 (m); 1.61 (m)	36.7	1.80 (m); 1.61 (m)	36.8
23	2.02 (m); 2.15 (m)	23.8	2.00 (m)	24.2	2.05 (m); 2.16(m)	23.8	2.05 (m); 2.14 (m)	23.8	2.06 (m)	24.3	2.06 (m)	24.1
24	5.13 (bt, 7.0)	126.1	5.10 (brt, 7.0)	125.9	5.11 (m)	126.1	5.12 (m)	126.1	5.11 (bt, 7.2)	125.9	5.11 (bt, 7.2)	126.1
25	-	132.2	-	132.3		132.2	-	132.2	-	132.3	-	132.2
26	1.68 (s)	25.9	1.68 (s)	25.9	1.68 (s)	25.9	1.68 (s)	25.9	1.69 (s)	25.9	1.69 (s)	25.9
27	1.62 (s)	18.0	1.62 (s)	17.9	1.62 (s)	18.0	1.62 (s)	18.0	1.62 (s)	18.0	1.62 (s)	18.0
28	0.86 (s)	28.6	0.82 (s)	28.5	0.82 (s)	28.5	0.77 (s)	28.4	0.83 (s)	28.6	0.83 (s)	28.6
29	1.10 (s)	17.7	1.08 (s)	17.8	1.07 (s)	17.79	1.02 (s)	16.7	1.07 (s)	17.9	1.07 (s)	17.9
30	0.92 (s)	17.4	0.92 (s)	17.2	0.91 (s)	17.3	0.91 (s)	17.4	0.89 (s)	17.1	0.89 (s)	17.3

**Table 2:** ^1^H- and ^13^C-NMR spectroscopic data of the sugar moieties of gypenosides CP1-6 (**1**–**6**).

No.	1		2		3		4		5		6	
	δ_H_	δ_C_	δ_H_	δ_C_	δ_H_	δ_C_	δ_H_	δ_C_	δ_H_	δ_C_	δ_H_	δ_C_
1′	4.42 (d, 7.5)	104.7	4.42 (d, 7.5)	104.6	4.41 (d, 7.8)	104.6	4.40 (d, 7.8)	105.2	4.42 (d, 7.8)	104.6	4.41 (d, 7.8)	104.6
2′	3.56 (m)	82.4	3.55 (m)	82.7	3.56 (m)	82.7	3.45 (m)	83.4	3.54 (dd, 7.8, 9.0)	83.2	3.55 (dd, 7.8, 9.0)	83.2
3′	3.58 (m)	78.7	3.58 (m)	78.7	3.58 (m)	78.7	3.54 (t, 9.0)	78.6	3.59 (m)	78.8	3.58 (m)	78.8
4′	3.37 (m)	70.9	3.36 (m)	70.9	3.36 (m)	70.9	3.32 (m)	71.2	3.36 (m)	70.9	3.36 (m)	70.9
5′	3.34 (m)	78.0	3.19 (m)	77.9	3.34 (m)	78.0	3.23 (m)	77.5	3.20 (m)	77.9	3.35 (m)	78.0
6′	3.85 (dd, 5.0, 12.0)	62.3	3.85 (dd, 5.0, 12.0)	62.3	3.86 (dd, 1.8, 12.0)	62.3	3.83 (dd, 1.8, 12.0)	62.7	3.86 (dd, 1.8, 12.0)	62.3	3.86 (dd, 1.8, 12.0)	62.3
	3.66 (dd, 5.0, 12.0)		3.66 (dd, 5.0, 12.0)		3.66 (dd, 5.4, 12.0)		3.65 (dd, 5.4, 12.0)		3.66 (m)		3.65 (dd, 5.4, 12.0)	
1″	4.71 (d, 7.5)	105.0	4.71 (d, 7.5)	105.2	4.70 (d, 7.8)	105.2	4.62 (d, 7.8)	105.5	4.71 (d, 7.8)	105.4	4.71 (d, 7.8)	105.4
2″	3.23 (m)	76.1	3.24 (dd, 7.5, 9.0)	76.1	3.24 (t, 8.4)	76.1	3.23 (t, 8.4)	76.4	3.24 (t, 8.4)	76.1	3.23 (t, 8.4)	76.1
3″	3.35 (m)	77.9	3.35 (m)	77.9	3.35 (m)	77.9	3.35 (m)	77.7	3.35 (m)	78.0	3.34 (m)	78.1
4″	3.29 (m)	71.4	3.32 (m)	71.4	3.32 (m)	71.4	3.32 (m)	71.3	3.33 (m)	71.4	3.33 (m)	71.3
5″	3.43 (m)	75.3	3.45 (m)	75.3	3.45 (m)	75.3	3.44 (m)	75.5	3.46 (dd, 1.8, 5.4)	75.2	3.46 (m)	75.3
6″	4.15 (dd, 5.0, 12.0)	65.0	4.18 (dd, 5.0, 12.0)	64.7	4.18 (dd, 4.8, 12.0)	64.7	4.18 (dd, 4.8, 12.0)	64.7	4.20 (dd, 5.4, 12.0)	65.2	4.20 (dd, 4.8, 12.0)	65.1
	4.31 (dd, 5.0, 12.0)		4.37 (dd, 5.0, 12.0)		4.36 (dd, 1.8, 12.0)		4.37 (dd, 1.8, 12.0)		4.38 (dd, 1.8, 12.0)		4.38 (dd, 1.8, 12.0)	
1a		172.8		168.1		168.1		168.2		138.4		138.4
2a	2.04 (s)	20.9	5.88 (dq, 1.5, 15.5)	123.6	5.88 (dq, 1.5, 15.6)	123.5	5.89 (dq, 1.8, 15.6)	123.5	7.38 (d, 7.2)	127.4	7.38 (d, 7.2)	127.3
3a			7.00 (dq, 7.0, 15.5)	146.6	7.00 (dq, 7.2, 15.5)	146.6	7.00 (dq, 7.2, 15.6)	146.6	7.29 (t, 7.2)	129.6	7.29 (t, 7.2)	129.7
4a			1.89 (dd, 1.5, 7.0)	18.1	1.89 (dd, 1.8, 7.2)	18.1	1.89 (dd, 1.8, 7.2)	18.1	7.20 (t, 7.2)	128.6	7.21 (t, 7.2)	128.6
5a									7.29 (t, 7.2)	129.6	7.29 (t, 7.2)	129.7
6a									7.38 (d, 7.2)	127.4	7.38 (d, 7.2)	127.3
7a									6.52 (d, 16.2)	134.6	6.52 (d, 16.2)	134.6
8a									6.33 (dd, 7.2, 16.2)	122.7	6.33 (dd, 7.2, 16.2)	122.7
9a									3.30 (m)	38.7	3.30 (m)	38.7
10a										173.4		173.4
1’’’	4.56 (d, 8.0)	98.1	4.60 (d, 8.0)	98.3	4.56 (d, 7.8)	98.1	4.56 (d, 8.4)	98.1	4.59 (d, 7.8)	98.3	4.56 (d, 7.8)	98.1
2’’’	3.12 (m)	75.3	3.08 (m)	75.4	3.11 (m)	75.3	3.11 (m)	75.3	3.07 (m)	75.4	3.12 (m)	75.3
3’’’	3.33 (m)	78.6	3.34 (m)	78.3	3.32 (m)	78.6	3.32 (m)	78.6	3.35 (m)	78.2	3.33 (m)	78.6
4’’’	3.31 (m)	71.4	3.32 (m)	71.2	3.32 (m)	71.4	3.32 (m)	71.4	3.33 (m)	71.2	3.32 (m)	71.4
5’’’	3.32 (m)	76.7	3.18 (m)	78.0	3.39 (m)	76.7	3.39 (m)	76.7	3.20 (m)	77.9	3.38 (m)	76.7
6’’’	3.73 (dd, 5.5, 11.5)	70.1	3.64 (dd, 5.5, 11.5)	62.5	3.72 (dd, 5.4, 11.4)	70.1	3.73 (m)	70.1	3.64 (m)	62.5	3.73 (dd, 5.4, 11.4)	70.1
	4.00 (dd, 2.0, 11.5)		3.77 (dd, 2.0, 11.5)		4.00 (dd, 2.4, 11.4)		4.00 (dd, 1.8, 11.4)		3.77 (dd, 2.4, 12.0)		4.00 (dd, 1.8, 11.4)	
1’’’’	4.30 (d, 7.5)	105.5			4.29 (d, 7.2)	105.6	4.29 (d, 7.8)	105.6			4.29 (d, 7.8)	105.6
2’’’’	3.20 (m)	74.8			3.20 (m)	74.8	3.19 (d, 7.2)	74.8			3.20 (d, 7.2)	74.8
3’’’’	3.30 (m)	77.5			3.30 (m)	77.5	3.29 (m)	77.5			3.30 (m)	77.5
4’’’’	3.47 (m)	71.2			3.46 (m)	71.2	3.47 (m)	71.2			3.46 (m)	71.2
5’’’’	3.18 (m)	66.8			3.18 (m)	66.8	3.18 (m)	66.8			3.18 (m)	66.8
	4.00 (dd, 2.0, 11.5)				3.84 (dd, 5.4, 11.4)		3.84 (dd, 5.4, 11.4)				3.84 (dd, 5.4, 11.4)	

**Table 3:** Antiproliferative data for compounds **1**–**25** against cancer cell lines.

Cmpd.	A549 Cell Line		HepG2 Cell Line		Number of Sugars
	Inhibition (%) ^a^	EC_50_ (μM)	Inhibition (%)	EC_50_ (μM)	
**1**	16.4 ± 3.97	(-) ^b^	38.9 ± 3.82	(-)	4
**2**	84.1 ± 7.97	59.4 ± 2.51	83.0 ± 3.91	60.4 ± 0.63	3
**3**	23.3 ± 6.20	(-)	44.0 ± 2.28	(-)	4
**4**	17.0 ± 2.00	(-)	46.4 ± 2.60	(-)	4
**5**	42.7 ± 1.41	(-)	71.1 ± 0.60	29.3 ± 0.26	3
**6**	29.7 ± 5.34	(-)	55.5 ± 6.45	54.2 ± 2.07	4
**7**	37.1 ± 0.78	(-)	53.8 ± 3.43	89.1 ± 3.75	3
**8**	6.5 ± 5.16	(-)	30.7 ± 5.32	(-)	3
**9**	14.5 ± 6.04	(-)	31.1 ± 7.76	(-)	4
**10**	20.9 ± 2.62	(-)	44.1 ± 1.96	(-)	3
**11**	94.4 ± 0.28	70.1 ± 2.34	93.1 ± 0.99	76.2 ± 2.10	2
**12**	27.8 ± 11.35	(-)	60.4 ± 6.34	100.7 ± 1.36	2
**13**	65.1 ± 7.29	87.3 ± 3.39	73.3 ± 1.81	68.4 ± 0.57	2
**14**	17.9 ± 2.72	(-)	24.5 ± 2.79	(-)	4
**15**	43.2 ± 3.67	73.8 ± 2.86	56.5 ± 1.55	75.4 ± 1.30	3
**16**	25.2 ± 1.35	(-)	37.3 ± 0.53	(-)	4
**17**	13.1 ± 2.88	(-)	11.5 ± 1.17	(-)	-
**18**	16.1 ± 5.32	(-)	9.4 ± 3.02	(-)	-
**19**	19.3 ± 1.40	(-)	32.5 ± 3.83	(-)	-
**20**	13.0 ± 3.40	(-)	18.0 ± 2.99	(-)	-
**21**	15.5 ± 2.73	(-)	29.2 ± 2.45	(-)	-
**22**	22.9 ± 12.78	(-)	26.6 ± 6.30	(-)	-
**23**	21.7 ± 9.46	(-)	31.2 ± 3.45	(-)	-
**24**	10.9 ± 6.06	(-)	25.2 ± 3.65	(-)	-
**25**	5.6 ± 3.12	(-)	6.5 ± 3.17	(-)	-

^a^ Inhibition (%) of pure compounds against cell lines at 100 μg/mL; ^b^ (-): ED50 > 100 g/mL.

## Supplementary Materials

The ^1^H- and ^13^C-NMR spectra of compounds **1**–**6** are available online.
