# Supplementary material for: New Dammarane-Type Saponins from Gynostemma pentaphyllum
Source: Molecules. 2019 Apr 8;24(7):1375. doi: 10.3390/molecules24071375 (PMC6480664; doi:10.3390/molecules24071375)
Supplement: Supplementary file 1 [file molecules-24-01375-s001.pdf]

Supporting Information

New Dammarane-type Triterpene Saponins from *Gynostemma pentaphyllum* (Jiaogulan)

Po-Yen Chen [1,2,†], Chih-Chao Chang [1,†], Hui-Chi Huang [3,†], Li-Jie Zhang [1], Chia-Ching Liaw [4], Yu-Chi Lin [1], Nham-Linh Nguyen [1,5], Thanh-Hoa Vo [1,5], Yung-Yi Cheng [6,7], Susan L. Morris-Natschke [6], Kuo-Hsiung Lee [6,7,*], and Yao-Haur Kuo [1,5,8,*]

[1] Division of Chinese Materia Medica Development, National Research Institute of Chinese Medicine, Taipei 112, Taiwan; peik35@gmail.com (P.Y.C.); changhcym@gmail.com (C.C.C.); lijizhang@hotmail.com (L.J.Z.); m8952612@hotmail.com (Y.C.L.); kuoyh@nricm.edu.tw (Y.H.K.)

[2] Department of Life Sciences and Institute of Genome Sciences, National Yang-Ming University, Taipei 112, Taiwan

[3] Department of Chinese Pharmaceutical Sciences and Chinese Medicine Resources, China Medical University, Taichung 404, Taiwan; hchuang@mail.cmu.edu.tw

[4] Department of Research and Development, Starsci Biotech Co. Ltd., Taipei 112, Taiwan; biogodas@hotmail.com

[5] The Ph.D. program in Clinical Drug Development of Chinese Herbal Medicine, College of Pharmacy, Taipei Medical University, Taipei 11031, Taiwan; nguyenlinhnham4201@gmail.com (N.L.N.); hoavo0808@gmail.com (T.H.V.)

[6] Natural Products Research Laboratories, Eshelman School of Pharmacy, University of North Carolina, Chapel Hill, NC 27599-7568, USA; susan_natschke@unc.edu (S.L.M.); khlee@unc.edu (K.H.L.); yungyi@email.unc.edu (Y.Y.C.)

[7] Chinese Medicine Research and Development Center, China Medical University and Hospital, Taichung 40402, Taiwan

[8] Graduate Institute of Integrated Medicine, College of Chinese Medicine, China Medical University, Taichung 404, Taiwan

* Correspondence: kuoyh@nricm.edu.tw (Y.H.K.), Tel.: +886-2-2820-1999 ext. 7061; yhlee@unc.edu (K.H.L.), +919-962-0066.

† These authors contributed equally to this work.

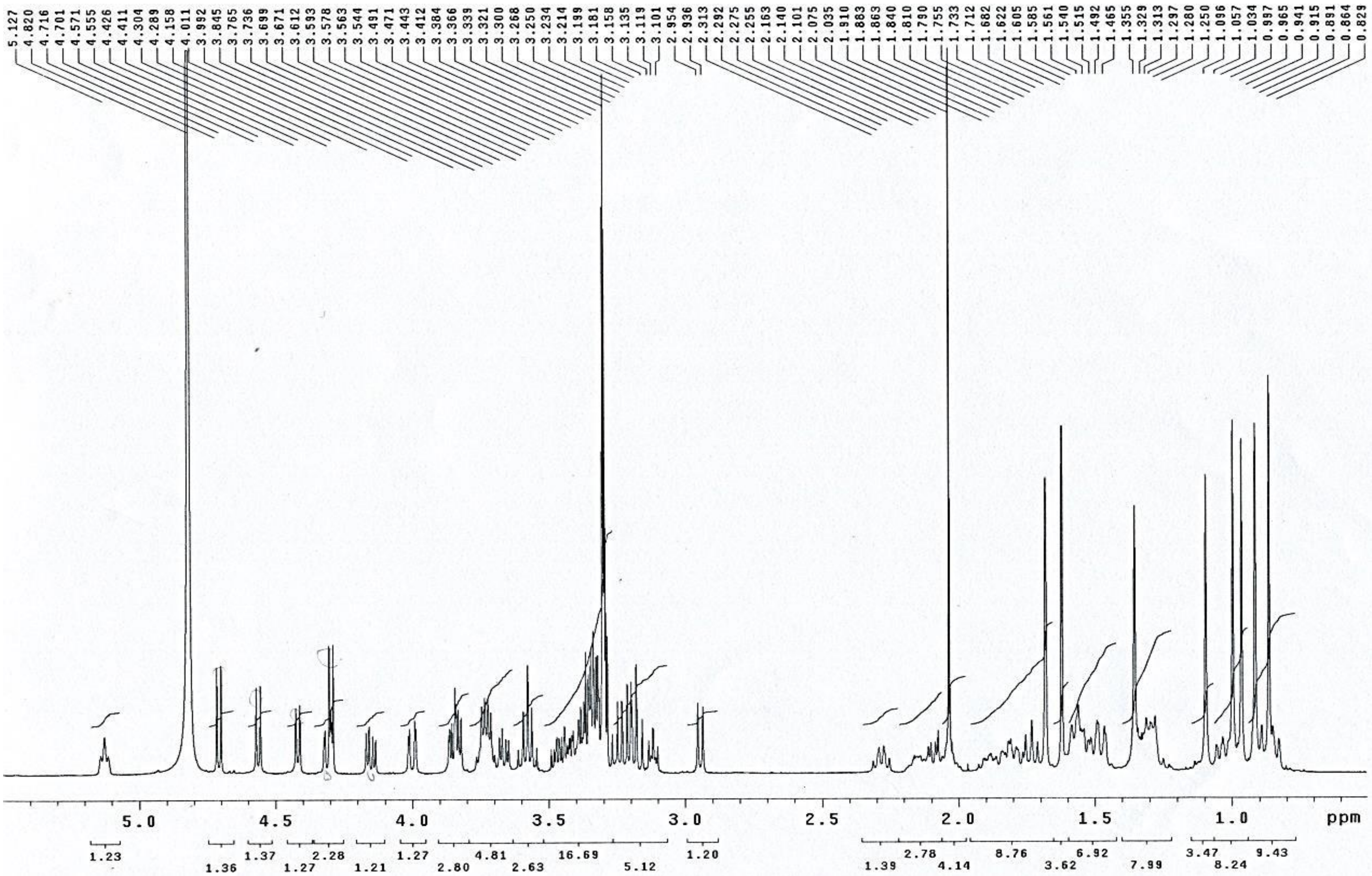

Figure S1. The ^{1}H-NMR spectrum of Gyenoside CP1 (**1**) (500 MHz in methanol-d_4)

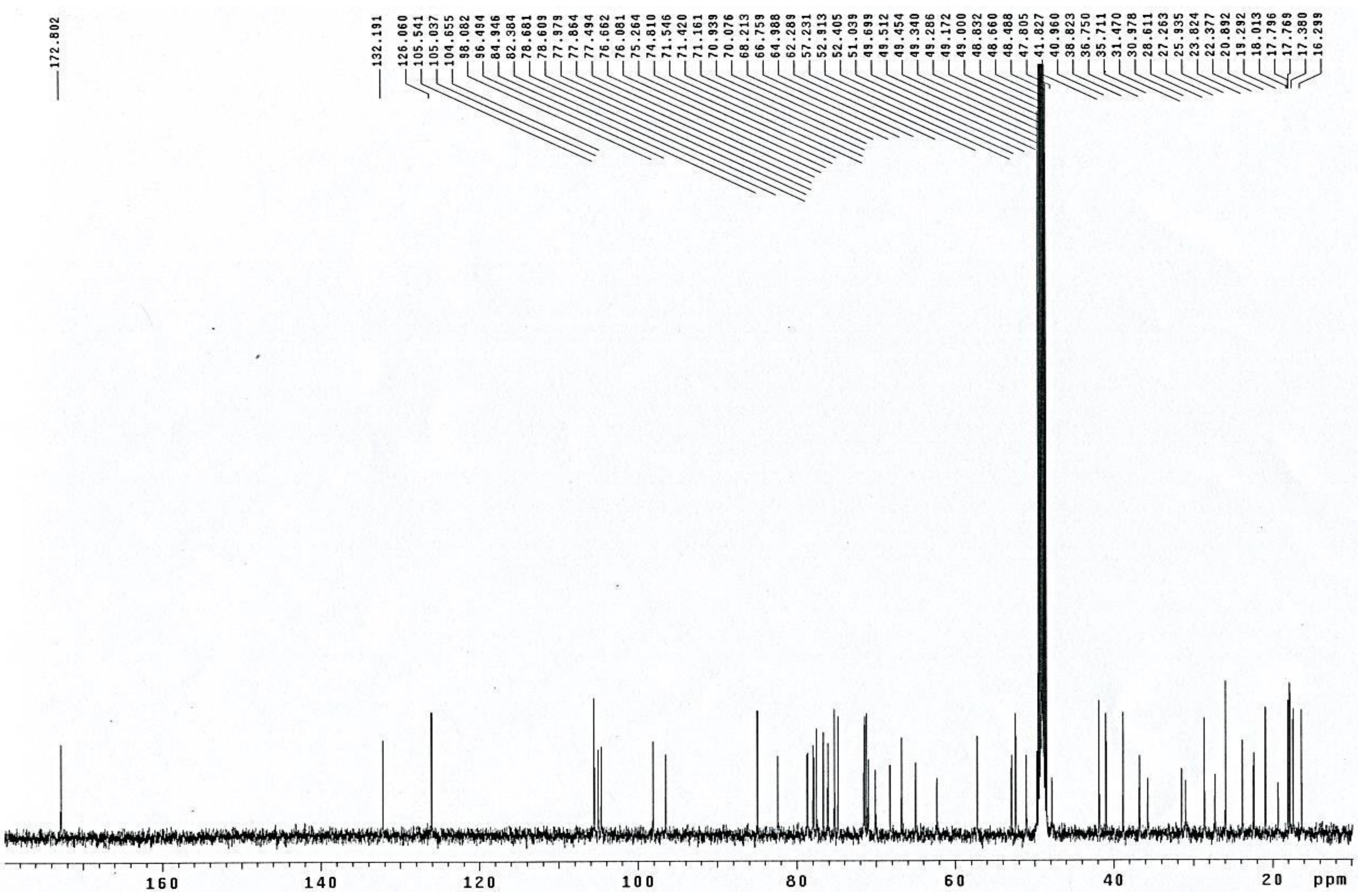

Figure S2. The ^{13}C-NMR spectrum of Gyenoside CP1 (**1**) (125 MHz in methanol-d_4)

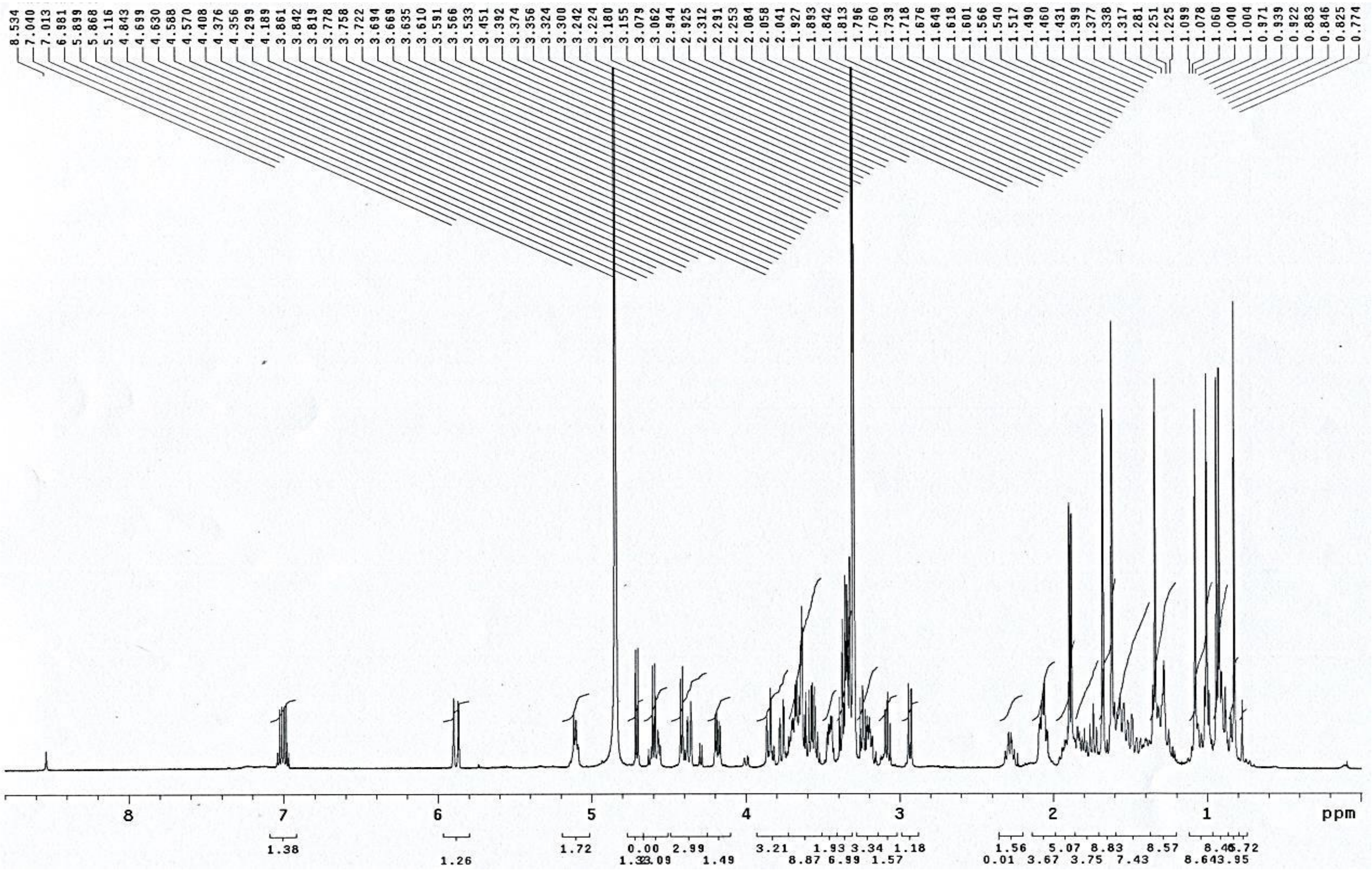

Figure S3. The ^{1}H-NMR spectrum of Gyenoside CP2 (**2**) (500 MHz in methanol-d_4)

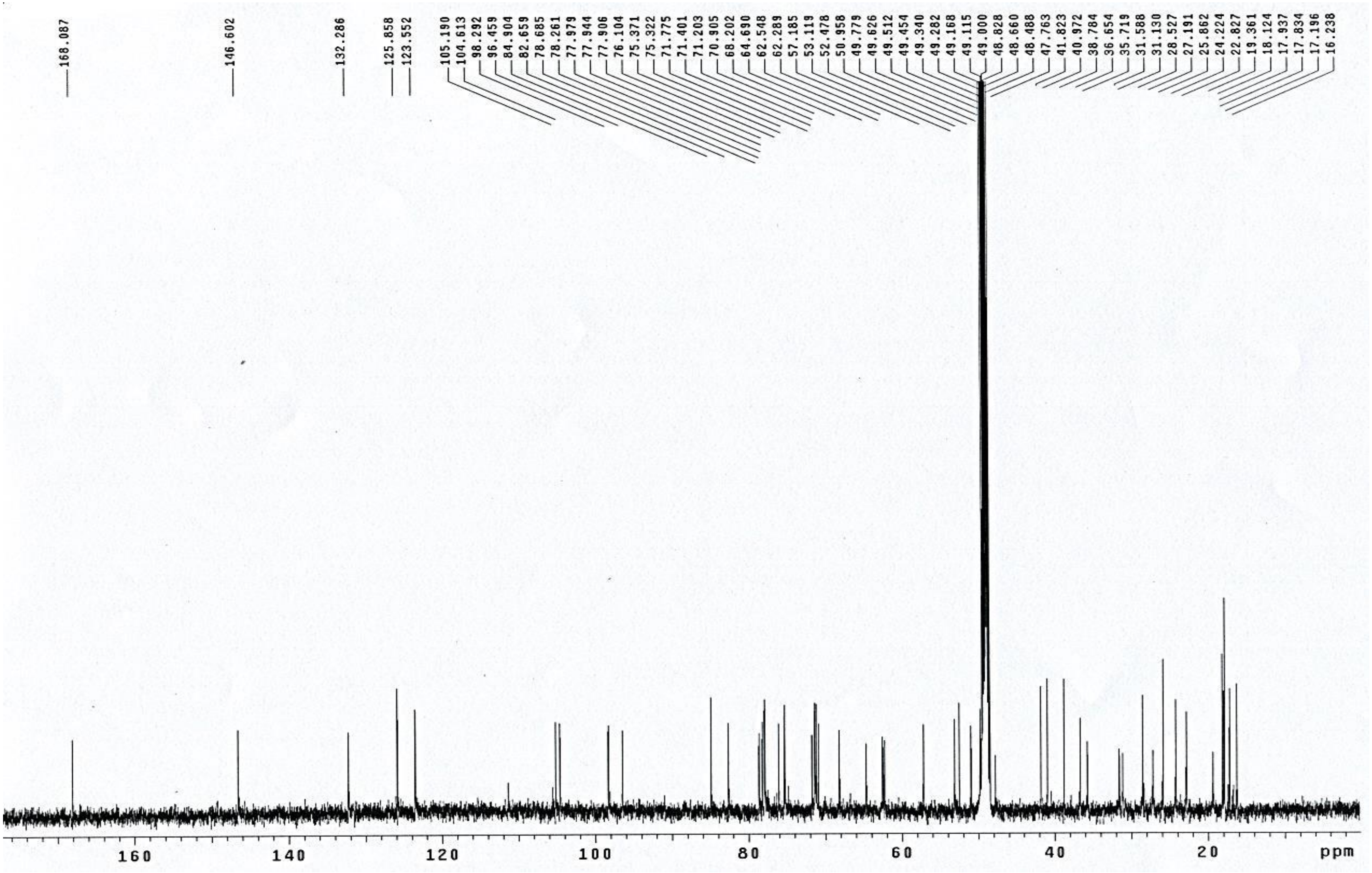

Figure S4. The ^{13}C-NMR spectrum of Gyenoside CP2 (**2**) (125 MHz in methanol-d_4)

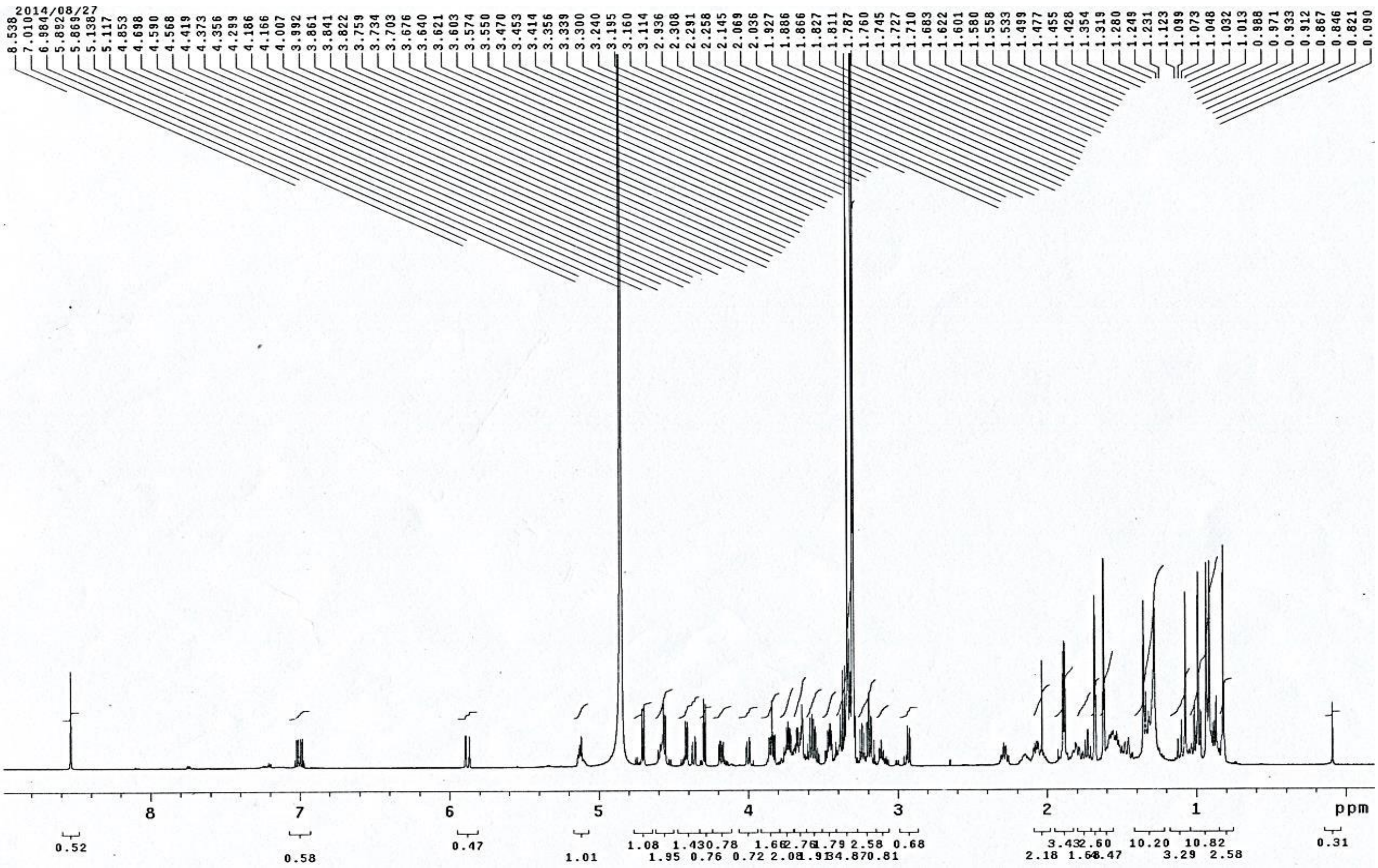

Figure S5. The ^{1}H-NMR spectrum of Gyenoside CP3 (**3**) (600 MHz in methanol-d_4)

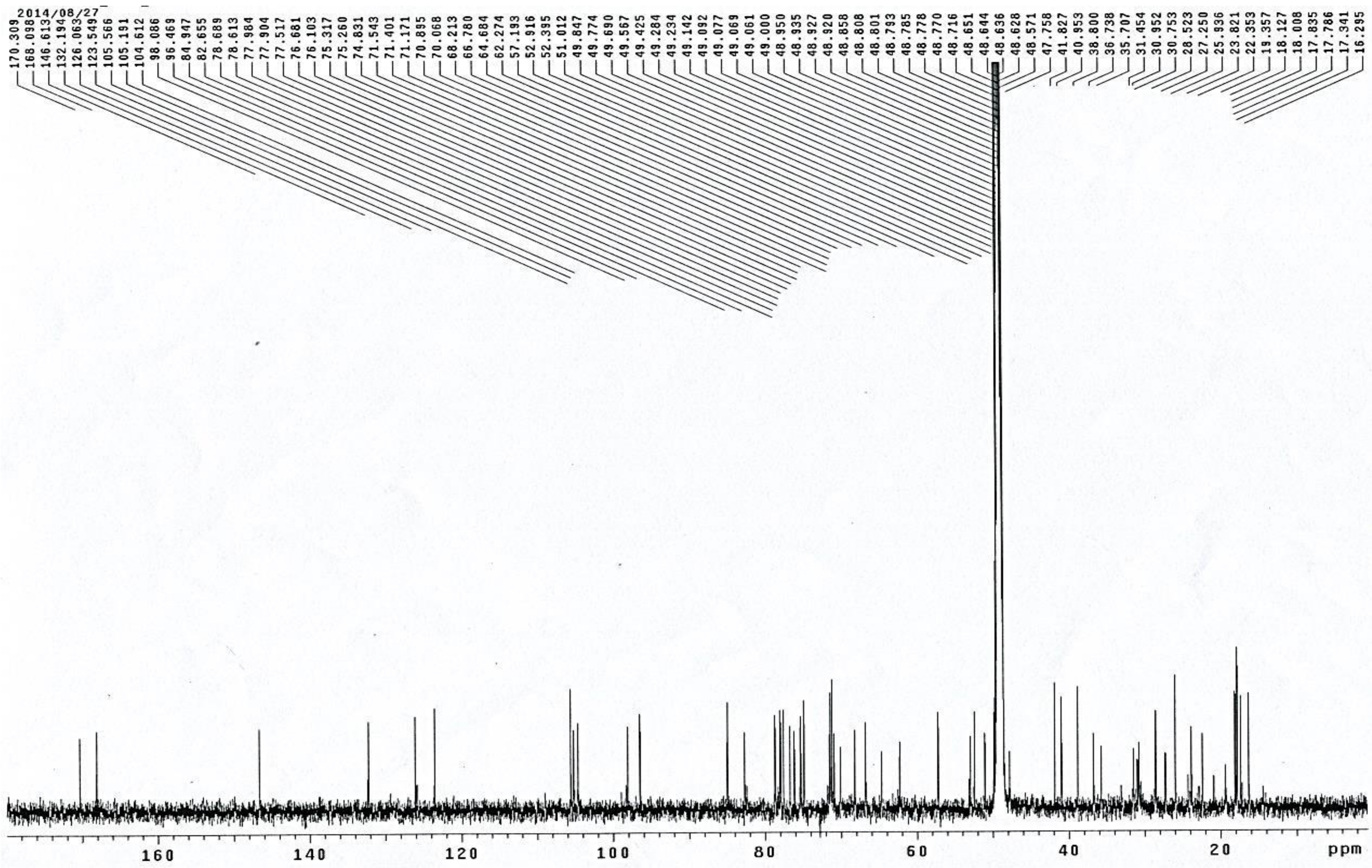

Figure S6. The ^{13}C-NMR spectrum of Gyenoside CP3 (**3**) (150 MHz in methanol-d_4)

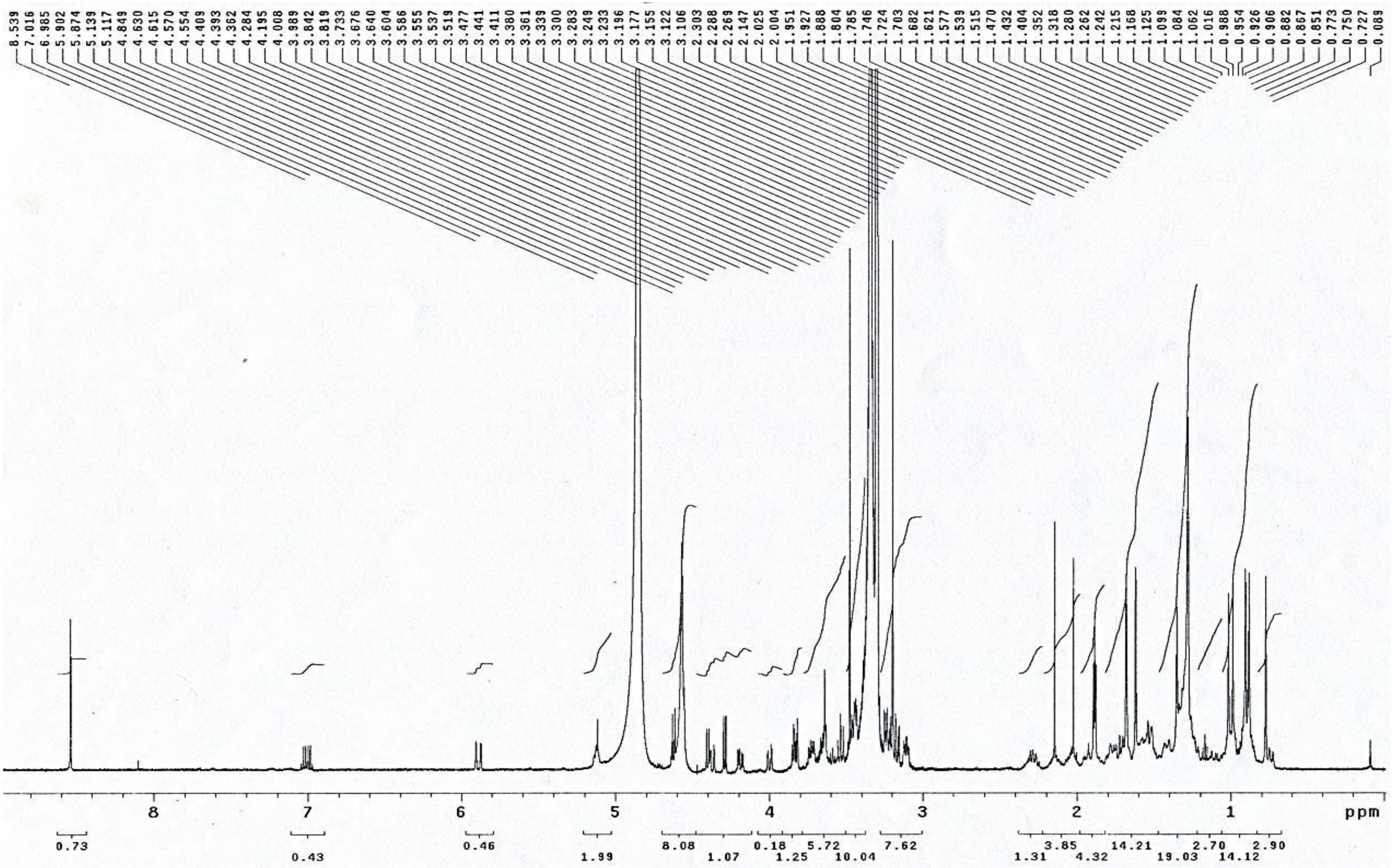

Figure S7. The ^{1}H-NMR spectrum of Gyenoside CP4 (**4**) (600 MHz in methanol-d_4)

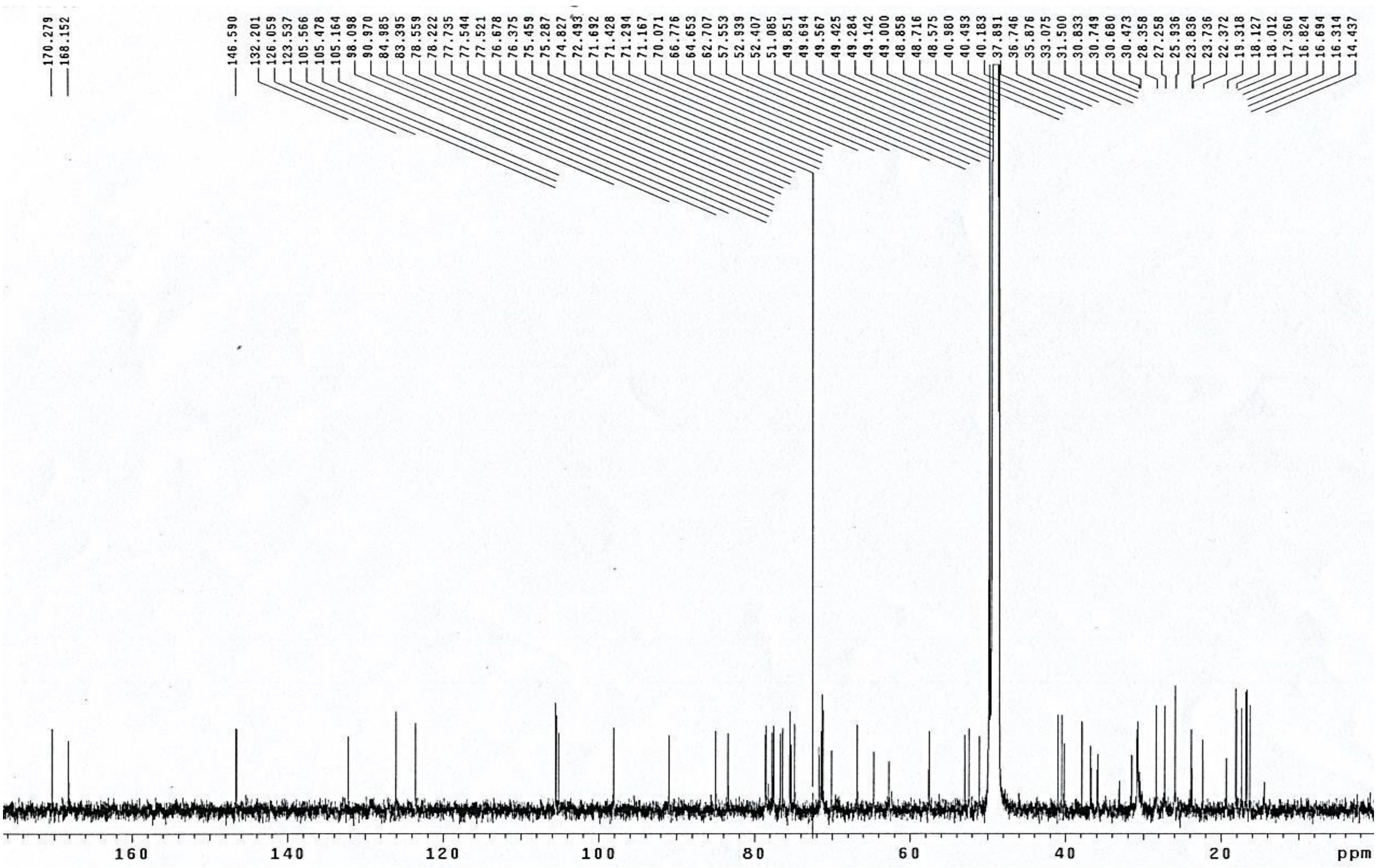

Figure S8. The ^{13}C-NMR spectrum of Gyenoside CP4 (**4**) (150 MHz in methanol-d_4)

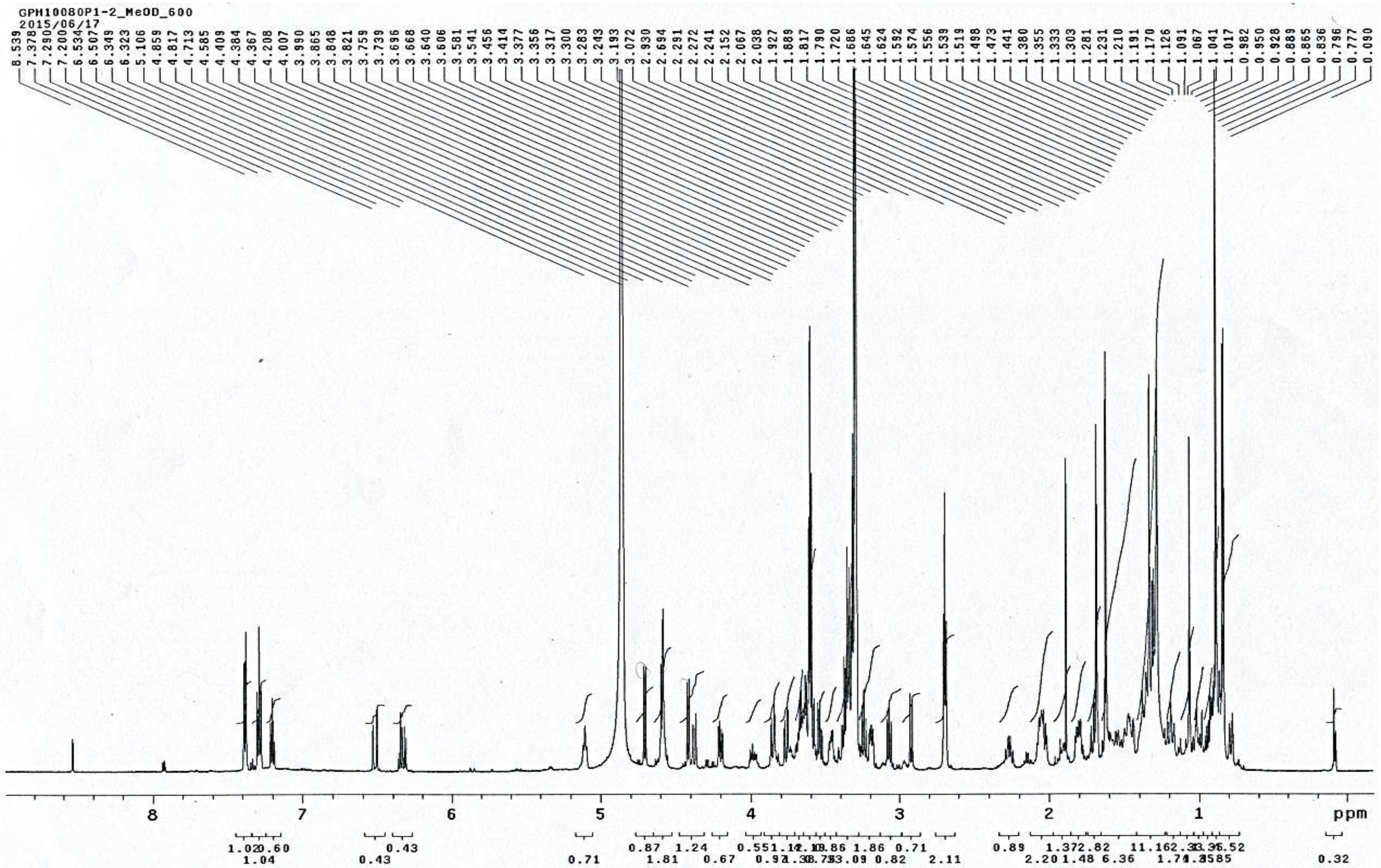

Figure S9. The ^{1}H-NMR spectrum of Gyenoside CP5 (**5**) (600 MHz in methanol-d_4)

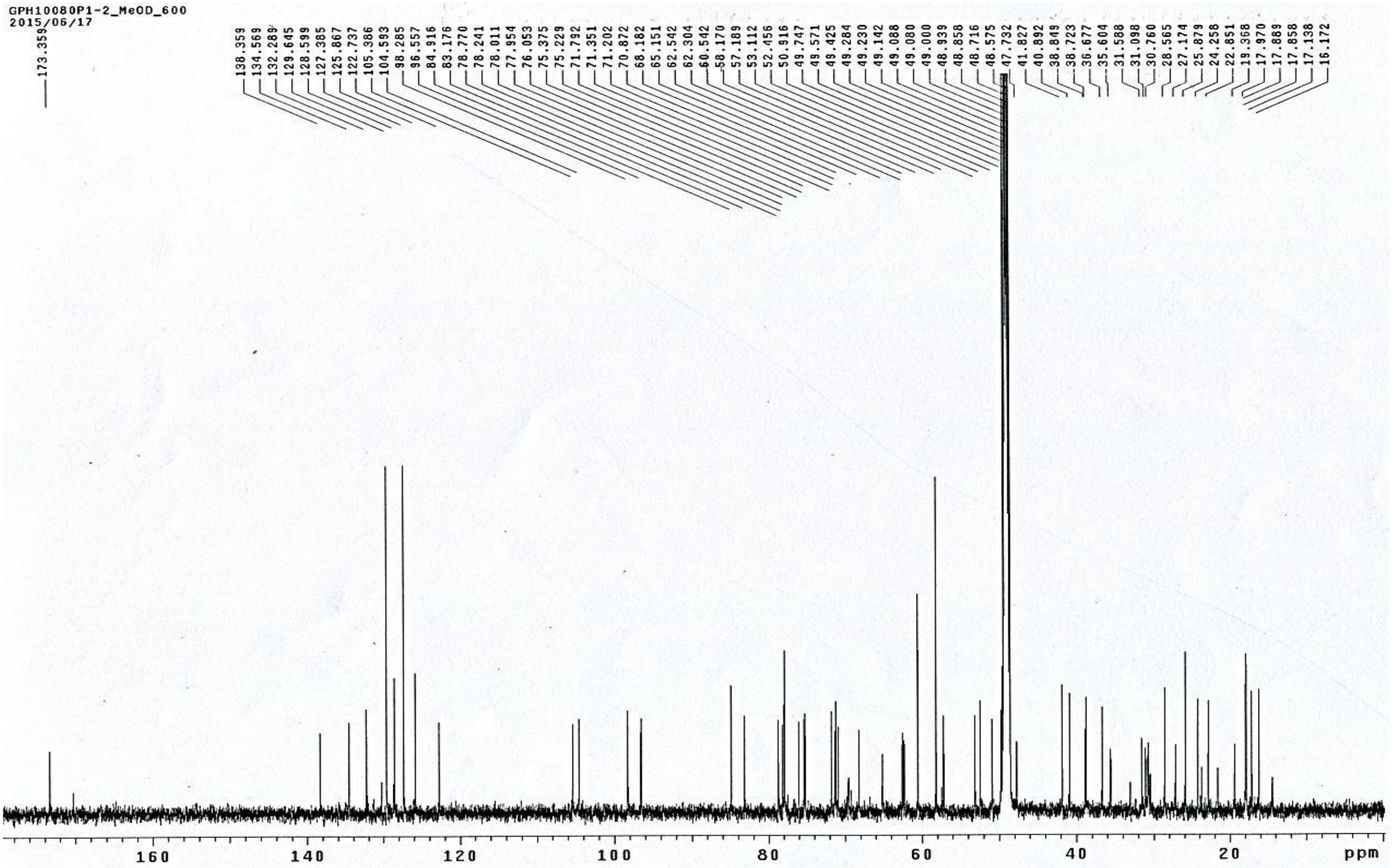

Figure S10. The ^{13}C-NMR spectrum of Gyenoside CP5 (**5**) (150 MHz in methanol-d_4)

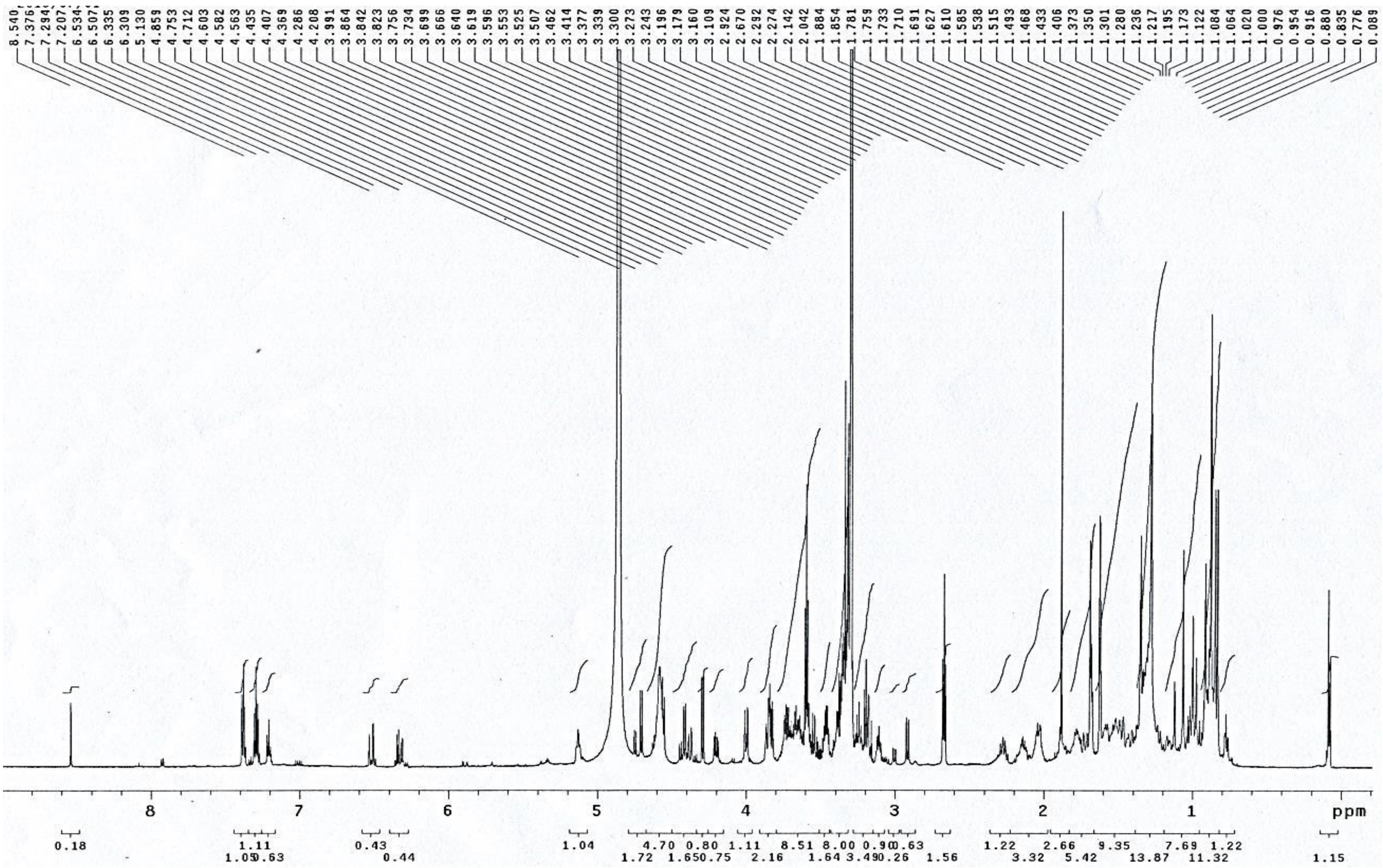

Figure S11. The ^{1}H-NMR spectrum of Gyenoside CP6 (**6**) (600 MHz in methanol-d_4)

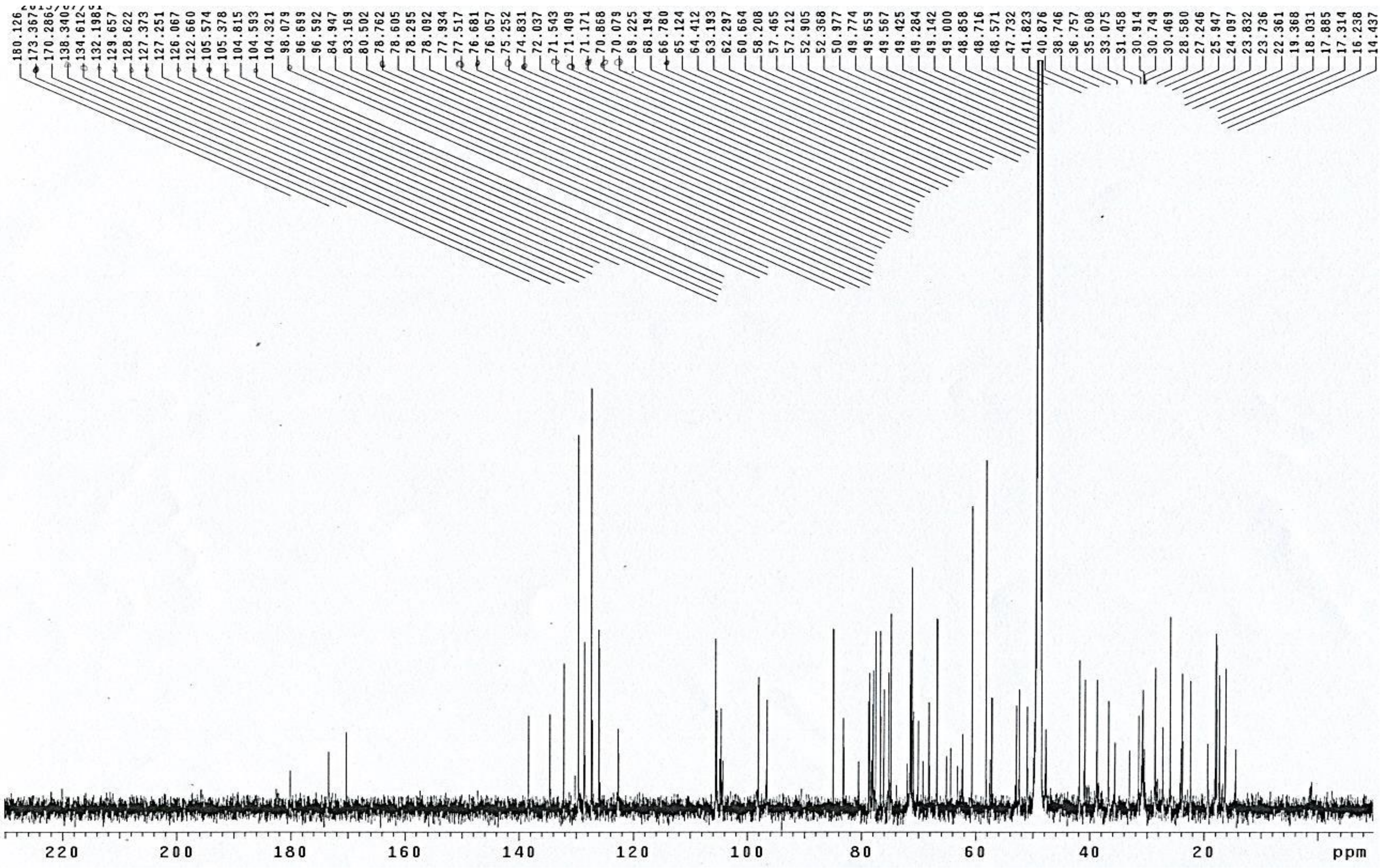

Figure S12. The ^{13}C-NMR spectrum of Gyenoside CP6 (**6**) (150 MHz in methanol-d_4)
